# Colicin N Binds to the Periphery of Its Receptor and Translocator, Outer Membrane Protein F

**DOI:** 10.1016/j.str.2007.12.023

**Published:** 2008-03-11

**Authors:** Thomas G. Baboolal, Matthew J. Conroy, Katrina Gill, Helen Ridley, Virak Visudtiphole, Per A. Bullough, Jeremy H. Lakey

**Affiliations:** 1 The Institute for Cell and Molecular Biosciences, Newcastle University, Newcastle-upon-Tyne, NE2 4HH, United Kingdom; 2 Department of Molecular Biology and Biotechnology, University of Sheffield, Sheffield S10 2TN, United Kingdom

**Keywords:** PROTEINS, SIGNALING

## Abstract

Colicins kill *Escherichia coli* after translocation across the outer membrane. Colicin N displays an unusually simple translocation pathway, using the outer membrane protein F (OmpF) as both receptor and translocator. Studies of this binary complex may therefore reveal a significant component of the translocation pathway. Here we show that, in 2D crystals, colicin is found outside the porin trimer, suggesting that translocation may occur at the protein-lipid interface. The major lipid of the outer leaflet interface is lipopolysaccharide (LPS). It is further shown that colicin N binding displaces OmpF-bound LPS. The N-terminal helix of the pore-forming domain, which is not required for pore formation, rearranges and binds to OmpF. Colicin N also binds artificial OmpF dimers, indicating that trimeric symmetry plays no part in the interaction. The data indicate that colicin is closely associated with the OmpF-lipid interface, providing evidence that this peripheral pathway may play a role in colicin transmembrane transport.

## Introduction

Protein translocation across membranes is a ubiquitous feature of biology and was once thought to require a water-filled pore to allow polar protein molecules across the hydrophobic bilayer. However, several models have been proposed recently whereby lipids play a critical role in the translocation pathway (Hessa et al., 2005; Rapaport, 2005; Slatin et al., 2002). Probably the most fundamental process is represented by the protein secretion apparatus known as Sec61 in eukaryotes, SecYEG in bacteria, and SecYEβ in archaea. In this example, unfolded polypeptides are translocated before folding (Robson and Collinson, 2006). Translocation of unfolded polypeptides reduces the minimum diameter of the pore required to shield polar polypeptide regions from the low dielectric constant of the membrane interior. Nevertheless, this pore must also deal with the insertion of hydrophobic helices of integral membrane proteins into the lipid bilayer (Rapoport et al., 2004). It appears to achieve this by a transient lateral opening of the pore, and, recently, strong evidence was obtained for the sorting of hydrophobic and amphipathic segments at a protein-lipid interface (Hessa et al., 2005).

Mitochondrial proteins are largely nuclear encoded and translocate across the outer membrane from the cytoplasm (Mokranjac and Neupert, 2005). This is accomplished by the TOM (translocase outer membrane) and TIM (translocase inner membrane) complexes (Rapaport, 2005). The β-barrel TOM complex provides a pore to deliver proteins across the outer membrane. Outer membrane β-barrel proteins are imported via the TOM pore into the intermembrane space and then inserted into the outer membrane by the SAM (sorting and assembly machinery) or TOB (topogenesis outer membrane β-barrel) complex (Paschen et al., 2005). This final step is similar to that in Gram-negative bacteria and involves at least one homologous protein, Omp85 (Gentle et al., 2005).

Mitochondria also import hydrophobic helical proteins into their outer membrane and do this in a TOM-dependent manner. Examples include those with single transmembrane stands, such as signal anchor proteins (Habib et al., 2003), and apoptosis regulators, such as Bcl (Rapaport, 2005), but possibly also multiple membrane-spanning proteins, such as liver carnitine palmitoyltransferase (Cohen et al., 2001), and viral proteins (Valentin et al., 2005). Because of the rigid β-barrel structure of the TOM pore, a mechanism for sideways release as in Sec is unlikely (Habib et al., 2003; Horie et al., 2003). Thus, it has been proposed that they insert via the protein-lipid interface at the periphery of the β-barrel Tom40 and possibly between several Tom40 dimers (Rapaport, 2005).

The only helical proteins known to reverse translocate across the Gram-negative bacterial outer membrane are toxic bacteriocins, such as the colicins of *Escherichia coli.* Colicins are 40–80 kDa proteins that kill cells closely related to the producer by translocating a large (15–25 kDa) toxic domain across the protective outer membrane. This domain is either a pore former or nuclease. The outer membrane normally acts as a molecular sieve permeable only to solutes smaller than 600 Da. Although large protein export pathways exist (Economou et al., 2006) and one colicin (E1) (James et al., 1996) does require TolC, (Koronakis et al., 2000) through which hemolysin toxins are exported (Holland et al., 2005), there is no evidence of a general link between colicins and dedicated protein export systems.

Because OmpF or a close homolog, such as PhoE or OmpC, is absolutely required for translocation of a number of colicins (Bourdineaud et al., 1990; Evans et al., 1996a; Fourel et al., 1990), their role in translocation has been discussed intensively (Bainbridge et al., 1998; Cao and Klebba, 2002; Kurisu et al., 2003; Vetter et al., 1998; Zakharov et al., 2004). Although OmpF is the translocator for most Tol-dependent (Lazdunski et al., 1998) colicins, most of which first bind a high-affinity receptor, such as BtuB (Cascales et al., 2007; Housden et al., 2005), Colicin N binds only to OmpF, which plays the role of both receptor and translocator (El-Kouhen et al., 1993). This simple complex may thus reveal how this protein acts as the general translocation route for many different colicins (Vetter et al., 1998). Experiments have clearly shown the blockage of OmpF ion channels by colicin domains (Stora et al., 1999; Zakharov et al., 2004, 2006) and the binding of colicin T domains to OmpF by isothermal titration calorimetry (Evans et al., 1996a; Housden et al., 2005). Nevertheless, we do not yet have conclusive evidence for the admittedly attractive and simple idea of a protein pore pathway through OmpF (Sharma et al., 2007; Vetter et al., 1998; Zakharov et al., 2004, 2006). It is well known that colicins unfold during translocation (Benedetti et al., 1992; Duché et al., 1994), but even elongated peptides exceed the diameter of the OmpF pore (Bainbridge et al., 1998; Cowan et al., 1992). Interestingly, TonB-dependent colicins (Lazdunski et al., 1998) seem only to require a high-affinity receptor (Buchanan et al., 2007).

Here, we describe the results of a combined biochemical and electron microscopy (EM) structural study indicating that colicin N binds to the outer surface of its receptor and translocator OmpF, displacing OmpF-bound LPS. The first helix of the pore-forming domain rearranges to allow binding to OmpF, which need not be in a trimeric conformation. Such an interaction with the periphery of OmpF thus raises the intriguing possibility that, as suggested for mitochondrial protein import, some part of the transmembrane translocation may occur at the protein-lipid interface.

## Results

### OmpF-Colicin N Complexes Form Ordered 2D Crystals

**Figure 1:**
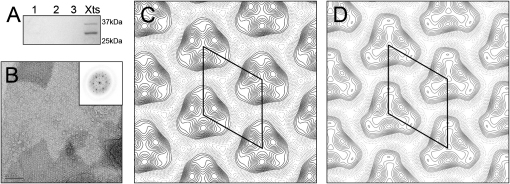
2D Crystals of OmpF + Colicin N-RP Are Visibly Different to OmpF Alone (A) Coomassie-stained SDS-PAGE of OmpF/colN-RP 2D-crystal (LPR, 1:2 w/w) together with several wash samples. (B) An electron micrograph showing an area of negatively stained OmpF/colN-RP 2D-crystal. Scale bar = 100 nm. The insert shows the relevant diffraction pattern. (C) Projection map showing the density derived from four merged OmpF/colN-RP crystals. The unit cell is indicated by the solid line. (D) Projection map showing the density derived from four merged OmpF 2D-crystals. The unit cell is indicated by the solid line.

Isolated complexes of colicin N with OmpF can be observed in negatively stained samples but, although they are clearly different from OmpF alone, they are currently of insufficient quality to contribute to a structural study (see the Supplemental Data available with this article online). The 2D crystallization of OmpF has been described elsewhere, and the dependence of lipid-to-protein ratio (LPR) on lattice structure was demonstrated (Dorset et al., 1983; Hasler et al., 1998). As a result of the difficulties in repeating and maintaining a precise LPR throughout detergent removal, several LPRs were evaluated (i.e., 0.8, 1.0, and 1.2). Vesicle structures of varying sizes were seen with all LPRs and appeared within 24 hr of dialysis. An LPR of 1.2 gave the best results with respect to size and crystal order. At lower LPRs, smaller vesicles were predominant, containing little or no ordered lattice. The crystals form in large vesicles (up to 5 μm in diameter) that collapse to form multiple-layers of 2D crystals, most of which were in register with each other. A construct consisting of the colicin N pore-forming and receptor binding domains (colN-RP) was used to form crystals of the complex, to avoid the influence of the unstructured translocation domain. Colicin N-RP/OmpF 2D crystals formed within 24 hr of dialysis in a two-fold molar excess of colN-RP. After washing to remove free protein, the crystals were analyzed for their protein content by SDS-PAGE. At each LPR (0.6, 0.8, 1.0, 1.2, and 1.4), both colN-RP and OmpF were present (Figure 1A). These crystals were similar to the OmpF-only crystals, with a diffraction pattern (Figure 1B) that confirms a hexagonal lattice and *p*3 symmetry consistent with previous OmpF crystals produced at these LPRs (Dorset et al., 1983; Hoenger et al., 1990). The best quality crystals were again seen at an LPR of 1.2. At other LPRs, the vesicles were smaller, with little or no ordered lattice. Image processing of the colN-RP/OmpF crystals gave a unit cell of a = b = 97.1 ± 0.6 Å (Figure 1C), slightly larger than that of the OmpF crystal (a = b = 93.6 ± 0.8 Å) (Figure 1D). Four separate images of each crystal type were merged in *p*3 symmetry to a resolution of 25 Å. The resulting OmpF map is consistent with previously published data (Dorset et al., 1983; Hoenger et al., 1990).

### Colicin N Is Located at the Periphery of OmpF Trimers

**Figure 2:**
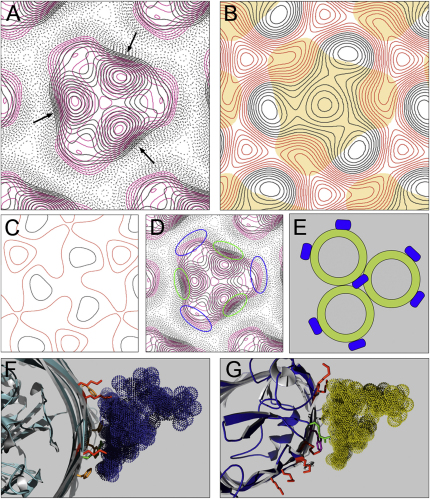
OmpF/ColN-RP Crystals Show Increased Peripheral Density at Monomer-Monomer Interfaces but Reduced Density where LPS Binds (A) Superposition of the merged and scaled projection maps from Figure 1 (OmpF crystal in magenta, OmpF/colN-RP crystal in black). The arrows indicate the areas of extra density contributed to the crystal by the presence of colN-RP. (B) A superposition of the OmpF footprint (solid orange) with difference map showing density due to colN-RP within the OmpF/colN-RP crystals. The colN-RP projection map was calculated from the subtraction of the merged and scaled OmpF data from that of the OmpF/colN-RP data in Fourier space. Negative contours are shown in red with positive contours shown in black. (C) A difference map showing the subtraction of two independently merged OmpF maps. Contours are at the same scale and orientation as in (B). (D) Superposition of the merged and scaled projection maps as in (A), with the areas of extra density in OmpF crystal indicated with blue and those of the complex in green. (E) A schematic of OmpF with bound LPS in those positions predicted by the work of Hoenger et al. (1990). The central LPS molecule on the trimeric axis of symmetry is not supported by more recent X-ray data, because no suitable cavity exists (Cowan et al., 1992). (F) FhuA with bound LPS (PDB code: 1QFG) (Ferguson et al., 2000). Indicated are those residues thought to constitute an LPS-binding motif (Lys in red, Arg in green, and Phe of the hydrophobic boundary in orange). (G) A proposed LPS-binding site located around Arg 235 based on the work of Ferguson et al. (2000) (Lys in red, Arg in green, Tyr in white, and Trp in purple; also see the Supplemental Data).

Comparison of the superposed, merged, and scaled projection maps from Figure 1 revealed some subtle differences between the two structures (Figure 2A). Because the crystallization methods were the same (i.e., the detergent and its concentration, lipid type and the LPR, buffer, and dialysis times), we conclude that the reproducible differences between the two structures in a series of samples are a result of colN-RP binding. A difference map was calculated by subtracting the merged and scaled OmpF map from that of the colN-RP/OmpF map in Fourier space to show features solely resulting from the presence of colN-RP (Figure 2B). This map reveals significant density extending from the external face of the OmpF barrel within the cleft between monomer-subunit interfaces of OmpF. This density almost certainly arises from bound colicin N-RP, which must interact with OmpF having a considerable proportion of the protein lying at the periphery of the OmpF envelope, possibly interacting with surrounding LPS.

### LPS Electron Density Is Removed by Colicin N

Areas of density at the outer edges of each monomer in the OmpF map are missing in the complex map (Figure 2D, blue circles). Disappearance of this density is manifest in the difference map by a slight negative density at the same location. This location has been proposed as an LPS-binding site on the basis of the 2D crystallization of purified OmpF-LPS complexes (Figure 2E; Hoenger et al., 1990), and it is likely that this loss of electron density indicates a possible displacement of LPS upon colN binding. Previous work on the outer membrane protein FhuA has identified a conserved LPS-binding motif (Ferguson et al., 2000). Interaction of 11 charged or polar residues with the negatively charged phosphate groups of the lipid A inner core and the diglucosamine were found and proposed to be responsible for the tight binding of LPS to FhuA. (Ferguson et al., 2000; Figure 2F). Of these 11 amino acids, four were found to be conserved between known LPS-binding proteins, which were identified using a structural search of the PDB (Ferguson et al., 2000). Colicins and outer membrane proteins, including OmpF, were also highlighted by the search (K. Diederichs, personal communication). By use of these data, a possible OmpF LPS-binding site is shown in Figure 2G comprising the conserved lysine and arginine residues of the LPS-binding motif. A similar site has been modeled onto the LPS-dependent outer membrane protease OmpT (Vandeputte-Rutten et al., 2001). The area indicated in Figure 2G shows good correlation with the areas of extra density found in the OmpF projection map (Figure 2D blue circles) and those found by Hoenger et al. (Hoenger et al., 1990) (Figure 2E). As a result of additional ion exchange purification steps, peripheral LPS molecules were not present in the detergent-solubilized OmpF X-ray structure (Cowan et al., 1992).

### Colicin N Displaces LPS from OmpF

**Figure 3:**
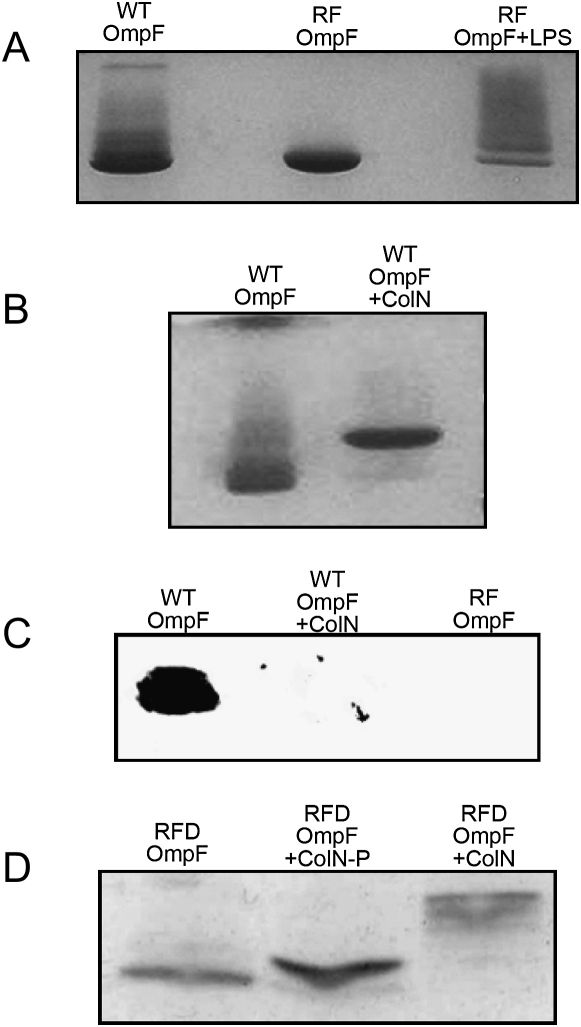
LPS Is Displaced from OmpF by Colicin N Complex Formation (A) The effect of LPS on the elecrophoretic migration of OmpF (WT OmpF, RF OmpF, and RF OmpF+LPS). (B) The effect of colN-RP on the electrophoretic migration of OmpF showing the shift in migration of OmpF owing to the increase mass of the complex and the loss of OmpF bound LPS. (C) Western blot using WN1 222-5 antibody (Di Padova et al., 1993) after SDS-PAGE to detect LPS. LPS is bound to OmpF but largely removed by colicin N addition, and the antibody shows no nonspecific binding to refolded LPS-free OmpF. (D) The effect of full-length colicin N (+ColN) and colicin P-domain (+ColN-P) on the electrophoretic migration of RFD (refolded dimeric) OmpF. The increase in mass of the RFD OmpF band is due to the increased molecular weight of the complex.

Without extensive ion exchange chromatography, LPS copurifies with OmpF, and it has also been shown to be critical in the assembly of outer membrane proteins in general (Bulieris et al., 2003; de Cock et al., 1999; Fourel et al., 1994). LPS associated with OmpF results in the formation of a “ladder/smear” upon SDS-PAGE because of differing numbers of LPS molecules associated with OmpF trimers (Holzenburg et al., 1989). It has been shown by free flow electrophoresis that four forms can be isolated—^lb^LPS (no loosely bound LPS), ^∗lb^LPS (1 molecule of loosely bound LPS per trimer), ^∗∗lb^LPS (2 molecules of loosely bound LPS per trimer), and ^+lb^LPS (8 molecules of loosely bound LPS per trimer). Each form had a defined homogenous mass measurable by SDS-PAGE and analytical ultracentrifugation. 2D crystals formed with ^+lb^LPS (as here) showed no effect of LPS upon the 2D lattice (Holzenburg et al., 1989). To demonstrate this further we used newly available, refolded trimeric OmpF (RF OmpF). This OmpF has been isolated from inclusion bodies and refolded in vitro to produce a fully folded, fully functional LPS-free trimeric OmpF (Visudtiphole et al., 2005). Figure 3A shows formation of the characteristic ladder on SDS-PAGE due to OmpF-associated LPS in both ^+lb^LPS (WT) OmpF purified from the outer membrane of *E. coli* BE3000 (Figure 3A, lane WT OmpF) and refolded trimeric OmpF with the addition of exogenous LPS from *E. coli* 0111:B4 (Figure 3A, lane RF OmpF+ LPS). These are both compared to the pure RF OmpF without LPS, which shows a single clear band (Figure 3A, lane RF OmpF). The slight difference seen in the migration patterns of WT OmpF and RF OmpF+LPS may be due to the use of a smooth LPS in the RF OmpF samples (Diedrich et al., 1990). Smooth LPS molecules contain the full oligosaccharide core and O antigen units and are therefore larger than those derived from rough strains (such as *E. coli* BE3000) and have been shown to bind preferentially to OmpF (Borneleit et al., 1989; Diedrich et al., 1990). WT OmpF/colN complex formation (Derouiche et al., 1996; Dover et al., 2000) results in the loss of the ladder effect, suggesting that LPS is displaced during complex formation (Figure 3B). Not only does complex formation appear to displace LPS, but it also results in dissociation of higher order OmpF structures/aggregates (Figure 3B). This effect is seen with all P-domain/OmpF complexes and also TolAII/OmpF complexes observable on SDS-PAGE (Derouiche et al., 1996; Dover et al., 2000). To determine whether the disappearance of the ladder on SDS-PAGE is due to removal of LPS, we used the anti-LPS antibody WN1 222-5 (Di Padova et al., 1993). No LPS could be detected in the complex formed by WT OmpF and colN or in RF OmpF, but a strong signal was observed in WT-OmpF alone (Figure 3C). To ensure that only the complex was present in the western blot, an excess of colN was used. Structural homology searches have revealed a possible LPS-binding site on colicin N (Ferguson et al., 2000), so we used fluorescently labeled LPS to detect whether LPS displaced from OmpF was bound by free colN. In an SDS-PAGE experiment where FITC-LPS was preincubated with RF OmpF, there was no fluorescence at the level of the free excess colN-RP. This experiment was inconclusive regarding LPS displacement from the complex, because free FITC-LPS migrated the same distance as OmpF (data not shown). Previously, the main role of LPS in colicin action was thought to be in the ability of long O antigen chains to inhibit both colicin and phage action on *E. coli* (Lakey et al., 1994; van der Ley et al., 1986) and possible interactions with Tol proteins (Cascales et al., 2007). Because the LPS is bound to the outer surface of the OmpF trimer, the current data indicate a clearer interaction of colicin N with this surface than has been previously proposed. The significant density from the EM study shows the colicin to be situated at the interface between two monomers in the trimer, but it has also been shown to bind dimeric OmpF that arises as a contaminant in normal preparations (Dover et al., 2000). Here, we made use of refolded dimeric OmpF, and our results confirmed (Figure 3D) that it also forms complexes with colicin N on SDS-PAGE. The dimer is asymmetric and is likely to form a structure resembling a trimer with a subunit missing so that the intermonomer interface is likely to remain (Visudtiphole et al., 2005). Thus, the binding site does not require a trimer but since we lack a folded monomer preparation this experiment cannot be taken to its natural conclusion.

### The First Helix of the Pore-Forming Domain Is Involved in Complex Formation

**Figure 4:**
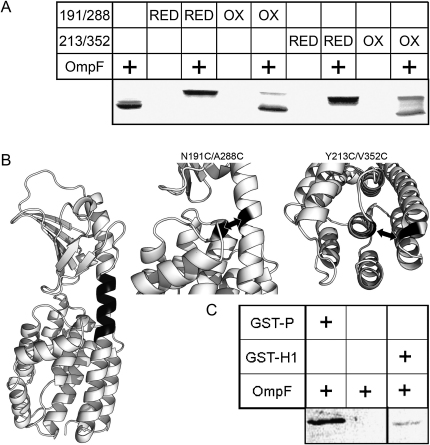
Complex Formation Involves Helix-1 of the Pore-Forming Domain (A) Coomassie-stained SDS-PAGE showing binding of the reduced (RED) and oxidized (OX) forms of colN N191C-A288C and colN Y213C-V352C to trimeric OmpF. Each disulfide fixes one end of Helix-1 in the native conformation. OmpF alone occurs as a doublet caused by LPS. Colicin/OmpF complex migrates at a higher MW, formation of which is inhibited by disulfide bond formation (OX). (B) Structure of colicin N (PDB code: 1A87) with the two disulfide bridges used in panel A represented with arrows and zoomed views. Dark region indicates the region of helix-1 fused to GST in GST-H1. (C) Anti-GST western blot showing the binding of OmpF to fusions of GST to the entire pore-forming domain GST-P or the first helix of the pore domain (GST-H1). GST-H1 was easily proteolyzed, causing the low intensity.

It was shown previously that the colicin P-domain and TolA-II (periplasmic domain) bind competitively to the OmpF trimer (Derouiche et al., 1996; Dover et al., 2000). TolA-II is a helical protein composed of 11 mer tandem repeats (Levengood et al., 1991), so it is straightforward to compare with likely sequences in colicin N. The most similar region is part of the N-terminal helix of the P-domain (ColN184–199). To test its involvement in complex formation, two disulfide bond mutants were designed that hold opposite ends of this helix in the conformation observed in the X-ray structure (Figure 4) (PDB code: 1A84) (Vetter et al., 1998). The mutant N191C-A288C, which binds the helix-1 (H1), was predicted by the program SSBOND (Hazes and Dijkstra, 1988) as having the correct geometry for a disulfide. However, in the absence of a useful prediction by SSBOND for the other end of H1, we chose Y213C-V352C, which links H1 to the tip of hydrophobic helix formed by H8/H9 with less favorable geometry (Figure 4). Each mutant showed shifts on SDS-PAGE upon oxidation, indicating disulfide formation (Supplemental Data), and was mixed with OmpF under both oxidizing and reducing conditions. For both cases, the formation of the disulfide bond inhibits complex formation, with N191C-A288C being more inhibitory than Y213C-V352C (Figure 4). Both mutants behaved as wild-type in the reduced state. Toxicity was tested on live cells in a fluorescent membrane depolarization assay (Bainbridge et al., 1998), and both mutants were inactive in the oxidized (disulfide) state. The addition of DTT allowed the mutants to regain their killing activity and, therefore, also confirms that the mutant Y213C-V352C does form a stable disulfide bond (Supplemental Data). Thus, conformational change of this region is required for complex formation with OmpF and toxicity. To further indicate the role of this region in complex formation, the entire P-domain and just the sequence K185-A195 were added to the C terminus of glutathione-S-transferase (GST) (Sharrocks, 1994). GST does not bind to OmpF in the SDS-PAGE assay, and an anti-GST western blot was used to detect interaction of the fusion proteins with trimeric OmpF. The GST-P-domain construct binds strongly, but the GST-colicin N (185–195) fusion (GST-H1) was easily proteolyzed. Nevertheless, the blot shows a clear binding imparted by this ten residue sequence (Figure 4).

## Discussion

Several groups of toxins are known to act by translocating proteins across membranes (Parker and Feil, 2005). In some examples, such as anthrax or cholera, a defined protein pore is created to insert a toxic subunit into the cytoplasm, but in diphtheria toxin, the translocon that transports the 270 residue catalytic domain is much less well defined. Colicin Ia has been shown to transport arbitrary cargo proteins, engineered onto its N terminus, across the lipid bilayer. This general transport system uses voltage to perform the seemingly impossible task of translocating folded charged proteins through a low dielectric barrier (Slatin et al., 2002). Furthermore, it has been proposed that combined protein-lipid or toroidal pores are formed by colicins in the inner membrane of *E. coli* (Sobko et al., 2004, 2006), by *E. coli* Hemolysin E (Tzokov et al., 2006) and by the eukaryotic channel-forming toxin Equinatoxin (Anderluh et al., 2003; Barlic et al., 2004). Thus, recent proposals for the involvement of lipid (Hessa et al., 2005; Rapaport, 2005), once considered “the last refuge of the intellectually bankrupt” (Qiu et al., 1996), have begun to suggest further alternatives to the protein-only model for transmembrane translocation pathways.

The translocation of Tol, but not Ton-dependent (Buchanan et al., 2007), colicins into Gram-negative cells requires either a trimeric porin (OmpF, OmpC, or PhoE) (Evans et al., 1996a) or TolC (Lazzaroni et al., 2002) and, thus, parasitizes host proteins not designed for protein import. The absolute requirement for these proteins leaves no doubt as to their central role in providing a pathway across the outer membrane. Isothermal titration calorimetry (ITC) measurements of colicin N binding to OmpF, OmpC, and PhoE showed that all three bound colicin with similar affinity, even though OmpF-bearing cells were much more sensitive. The difference in toxicity must therefore be due to differences in translocation. OmpF binds colicin N with a much larger enthalpic component, which is compensated by a significant entropic penalty; thus, efficient colicin translocation by OmpF correlates with unique colicin N-binding thermodynamics. Such binding is observed only when using full-length colicin N (Evans et al., 1996a, 1996b), and it has recently been demonstrated by ITC that the flexible translocation domain of colicin E9 binds specifically to OmpF (Housden et al., 2005). Because this domain also binds a periplasmic receptor (TolB), it is likely that it interacts with OmpF on its periplasmic face (Housden et al., 2005). Thus, complexes of pore-forming colicins with OmpF can require interactions with all three domains—translocation (Evans et al., 1996a), receptor (Evans et al., 1996b), and pore forming (Dover et al., 2000).

**Figure 5:**
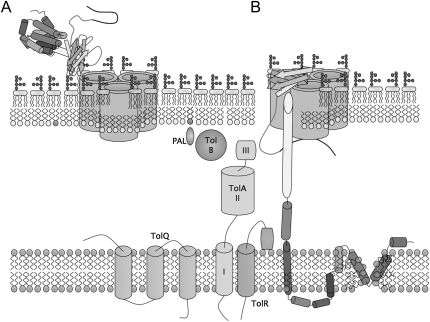
Possible Arrangement and Translocation Mechanism for Colicin N (A) A schematic representation of initial interaction of the colicin N receptor binding domain with OmpF in the *E. coli* outer membrane. (B) The suggested arrangement of unfolded colicin N according to data from this study. The pore-forming domain unfolds and interacts with the external surface of OmpF, filling the cleft between two monomers to agree with EM density while displacing LPS. The unfolded pore-forming domain is sufficient to make the ion channel, whereas the suggested rearrangement of helix-1 would be prevented by the disulfide bonds that prevented complex formation.

Ion channel measurements in artificial lipid membranes also reveal OmpF interactions with the R domain (Stora et al., 1999) and T domain (Zakharov et al., 2004) of colicin N by observation of transient blocking of the pore. The blocking by T domain occurs on one side of OmpF, but whether this is the extracellular (Zakharov et al., 2004) or periplasmic side (Danelon et al., 2003), as is likely from the biology (Housden et al., 2005), is not clear. Mutations in OmpF that affect colicin N binding are on the outer loops (E285,G285) or in the pore lumen (G119D) (Fourel et al., 1993; Jeanteur et al., 1994), and it is the latter, deep inside the pore, that conflicts most with a possible exterior route for protein translocation. However, this mutation is a true receptor-binding mutant whose effects are overcome under low-salt receptor bypass conditions where the role of OmpF is purely a translocator (Jeanteur et al., 1994). The narrow “eyelet” region of the OmpF pore is probably too small to accommodate a polypeptide, and OmpF unfolding would need to provide a suitable pore size such as that found in the anthrax toxin (Krantz et al., 2005). Disulfide bond mutants, which prevent localized eyelet unfolding, have no effect upon translocation and thus argue against the pore route (Bainbridge et al., 1998), although there are arguments supporting the classical model (Cao and Klebba, 2002). Studies using OmpF/OmpC chimeras show that translocation of colicin N by OmpF is dependent on residues 143–262 (Fourel et al., 1990), which form the outer wall of the β-barrel (Supplemental Data), are separated from the pore by the invaginated loop3 and coincide with the proposed LPS-binding site (Figure 2F and Supplemental Data). Importantly, both colicin N and C termini of colicin need to gain access to the periplasmic space through the outer membrane barrier for toxicity to occur. The evidence here is that the unfolded C-terminal domain inserts in clefts at the periphery of OmpF with direct binding by its first helix. The remaining helices are sufficient to span the periplasm and form a functional toxic pore (Baty et al., 1990; Figure 5). It is not clear where the N-terminal translocation domain fits in the current proposal. Finally, because colicin activity relies on the Tol-Pal complex, which has recently been shown to be part of the cell division machinery (Gerding et al., 2007), the OmpF employed by colicins may be newly synthesized. The relevance to the majority of colicins, which also bind to a high-affinity receptor, is best appreciated by examination of the X-ray crystal structure of the receptor complex of colicin E3 (Kurisu et al., 2003) and of the detailed model for OmpF recruitment provided by work on colicin E9 (Housden et al., 2005). The initial receptor-bound structure may thus present the N-terminal disordered domain for OmpF binding and the C-terminal toxic domain for translocation in a format comparable to that shown here.

In conclusion, we have revealed by electron crystallography the first, to our knowledge, visualization of a colicin within a membrane translocon. By such direct imaging and indirect biochemical methods, we show that colicin N makes intimate contact with the exterior of its translocator, displacing tightly bound lipid as it does so. Furthermore, we measured the interaction of helix-1 with OmpF, which was predicted in most models of group A colicin translocation (Cascales et al., 2007; Vetter et al., 1998). These discoveries argue strongly for the transmembrane translocation of colicins at the protein-lipid interface. Together with recently published evidence for protein translocation at other protein-lipid interfaces (Hessa et al., 2005; Rapaport, 2005), our data question the general assumption that protein translocation across membranes occurs exclusively though protein pores.

## Experimental Procedures

### Protein Expression and Purification

The Colicin N-RP construct was created using Quick Change mutagenesis to “loop-out” the translocation domain (residues 1–81) of the full-length gene. The mutagenic primer contained a 5′ region complementary to the MCS of the pET8c and the 3′ complementary half of the colicin N receptor-binding domain (the underlined half being complementary to the start of the receptor-binding domain [5′-CATCACCATCACTCGAGCAGTGCTAAGGTTGGAGAG-3′]). The Quick Change product thus lacked the translocation domain. All colicin constructs were expressed using the modified pET8c vector giving N-terminal six histidine tag (Politou et al., 1994). Expressed protein from *E. coli* BL21 pLysE was then purified using Ni-NTA affinity resin (Fridd et al., 2002). WT OmpF was extracted from the outer membrane of *E. coli* BE3000, as described elsewhere (Lakey et al., 1985). Refolded trimeric and dimeric OmpF was purified from inclusion bodies, as described in Visudtiphole et al., 2005.

### Preparation of OmpF/Colicin N Complexes in Detergent for Negative-Stain Electron Microscopy

Each complex was formed in a 2-fold molar excess of colicin in the presence of SDS (0.1% w/v) and was incubated for 30 min at 37°C. These complexes were applied to glow-discharged, carbon-coated grids and stained with uranyl acetate (2% w/v). Micrographs were recorded at 100 kV on a Philips CM100 EM onto Kodak Electron Image Film, SO163.

### Preparation of 2D Crystals for Negative-Stain Electron Microscopy

Formation of the 2D crystals followed the method developed by Dorset et al. (1983). WT OmpF purified in SDS from the outer membrane was buffer exchanged into 2D crystallization buffer (20 mM HEPES [pH 7.0], 10 mM MgCl_2_, 100 mM NaCl, 0.2 mM EDTA, 0.2 mM DTT, and 3 mM NaN_3_) supplemented with octyl-POE (1.0% v/v). Where required, colN-RP was added at a molar ratio of 1:2 (monomeric OmpF:colN-RP) and was incubated for 30 min at 37°C. To this, DMPC (Avanti Polar Lipids Inc., Alabaster, AL; 20 mM Tris [pH 7.5] and 1% [v/v] octyl-POE) was added at the relevant LPR (w/w). After mixing, the samples were incubated for a further 30 min at 37°C.

Dialysis of the mixture using a 3,500 MWCO Float A Lyzer (Spectrum Laboratories Ltd., Rancho Dominguez, CA) was performed at 37°C against 2D-crystallization buffer for at least 20 hr (all buffers were pre-equilibrated at 37°). After 50% of the dialysis buffer was replaced with fresh 2D-crystallization buffer, dialysis continued for a further 20 hr at 37°C. A further 50% of the dialysis buffer was then changed for Nano-pure water and dialyzed for a further 4 hr. This step was repeated three times. The sample was then incubated on ice for 10 min before being centrifuged at 2000× g for 5 min. Resuspension of the sample into equal volumes of Nano-pure water was followed by centrifugation at 2000× g for 5 min. This step was repeated, and SDS-PAGE was used to determine the presence of protein in the final crystals. Samples were negatively stained with uranyl formate (0.75% w/v).

### Image Processing

Micrographs were recorded at 100 kV on a Philips CM100 electron microscope equipped with a 1024 × 1024 CCD camera. Images of crystals that showed good diffraction were processed to a resolution of 25 Å, as described elsewhere (Crowther et al., 1996; Henderson et al., 1986). Symmetry analysis was performed using ALLSPACE (Valpuesta et al., 1994), and four separate images of each type of crystal merged in *p*3 symmetry. Difference maps were calculated by subtraction of the Fourier terms after first scaling amplitudes to yield equal total amplitude for each data set (Kubalek et al., 1987).

### SDS-PAGE Gel Shift Assay

All gel shift assays were performed on 12% (w/v) SDS-PAGE, as described elsewhere (Dover et al., 2000). Complex formation was achieved by incubating samples at 37°C for 30 min in the presence of SDS (0.1% w/v). Samples were analyzed without heat denaturation.

## Supplemental Data

Document S1. Five Figures and Supplemental References
