# Supplementary material for: Colicin N Binds to the Periphery of Its Receptor and Translocator, Outer Membrane Protein F
Source: Structure. 2008 Mar 11;16(3-24):371–9. doi: 10.1016/j.str.2007.12.023 (PMC2581486; doi:10.1016/j.str.2007.12.023)
Supplement: Document S1. Five Figures and Supplemental References [file mmc1.pdf]

Structure ***16***

Supplemental Data

Colicin N Binds to the Periphery of Its Receptor and Translocator, Outer Membrane Protein F

Thomas. G. Baboolal, Matthew J. Conroy, Katrina Gill, Helen Ridley, Virak Visudtiphole, Per A. Bullough, and Jeremy H. Lakey

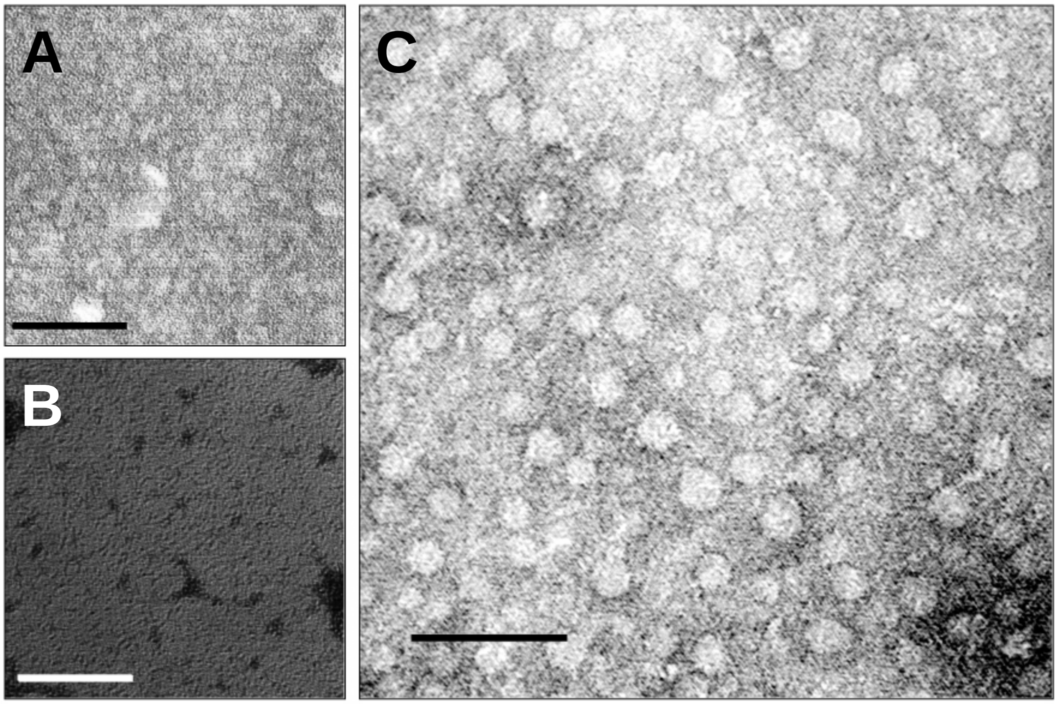

Figure S1. Colicin N and OmpF Form a Defined Complex in SDS

Electron micrographs of negatively stained detergent-solubilised (**A**) ColN-P, (**B**) OmpF and (**C**) OmpF/colN-P complex. Scale bar: 100nm. Complex was formed in a molar excess of colicin N pore forming domain (colN-P) at 37°C for 30 minutes in the presence of SDS (0.1 % w/v).

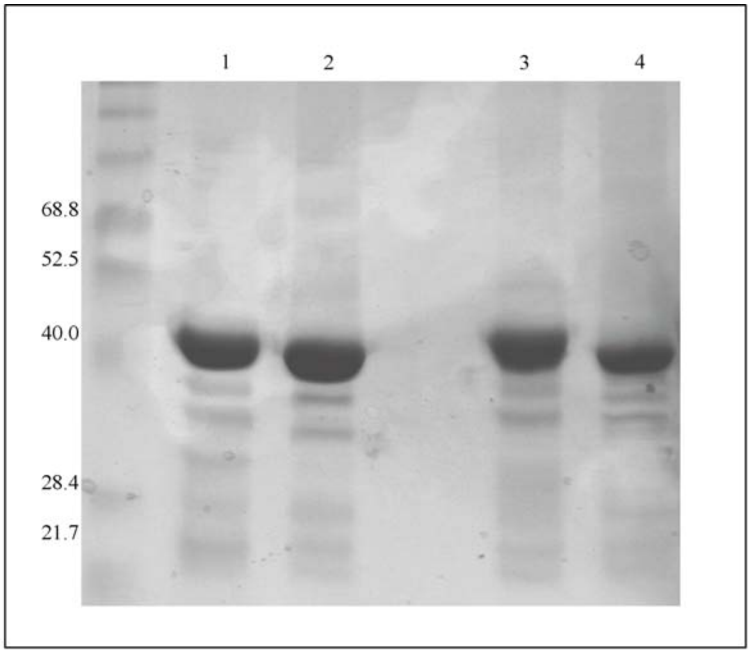

Figure S2. Oxidised and Reduced Forms of the Disulphide Bond Mutants

SDS PAGE of N191C-A288C (1) Reduced (2) Oxidised and Y213C-V352C (3) Reduced (4) Oxidised. In each case the oxidised mutant migrates further.

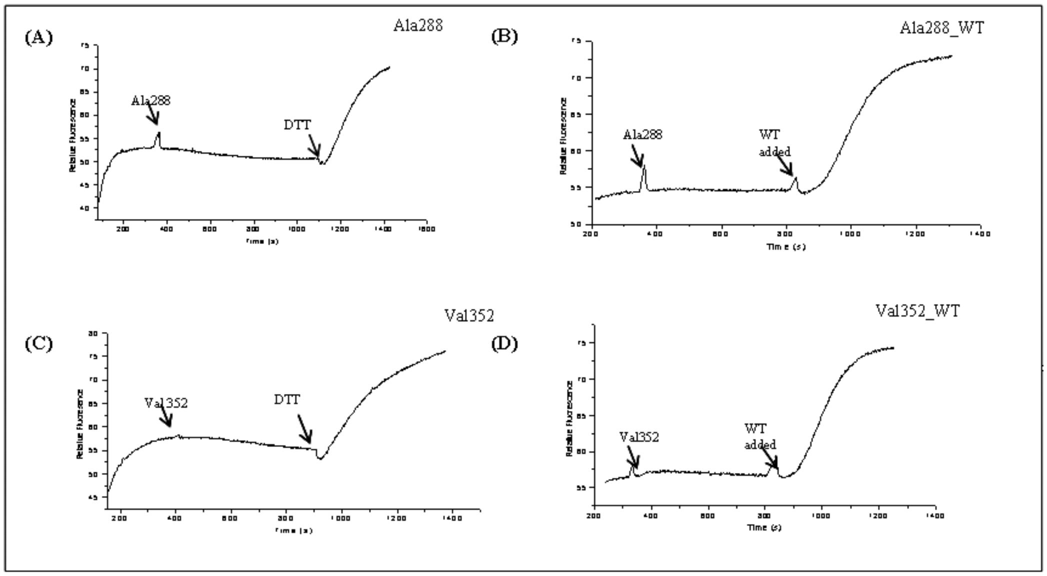

Figure S3. Depolarisation of *E coli* Cells by Colicin N Disulphide Mutants

Vertical scale is in arbitrary units of ANS fluorescence which increases when depolarisation of *E coli* BE3000 cell membranes by active colicin occurs (Bainbridge *et al*., 1998). **(**A) and (C) N191C-A288C and Y213C-V352C respectively. Arrows indicate addition of colicin and DTT. The lack of effect before and the rise in fluorescence after DTT addition indicates that both mutants are inactive in the oxidised (disulphide bonded) states and that the disulfide bonds form at near to 100% efficiency. **(**B) and (D) N191C-A288C and Y213C-V352C respectively in the oxidised state followed by Wild Type colicin N show no reduction in activity indicating that disulphide bonded and trapped colicins do not block the translocon.

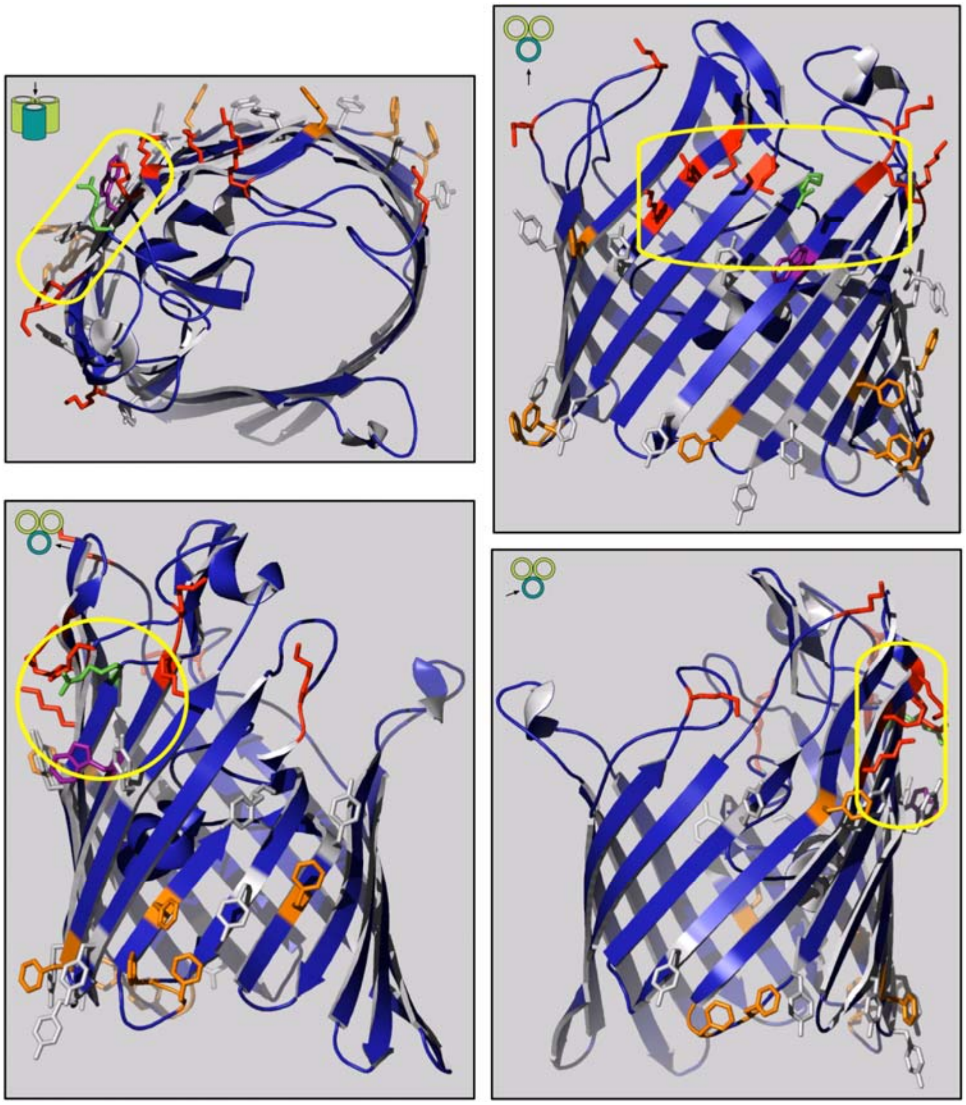

Figure S4. LPS Binding Site of OmpF

The putative LPS binding site on OmpF (Yellow box) was based upon the LPS binding site of FhuA (Ferguson *et al*., 2000). The site is located around Arg 235 (green) located above the ring of aromatic residues (which mark the limit of the hydrophobic membrane core) and flanked by Lys 209, 210, 277, 279 and 281 (Red). Aromatic ring residues are marked Phe (orange), Tyr (white), Trp (purple). For clarity only one OmpF monomer is shown and only outwardly pointing residues shown. The direction of each view with respect to the trimer is shown by the schematic.

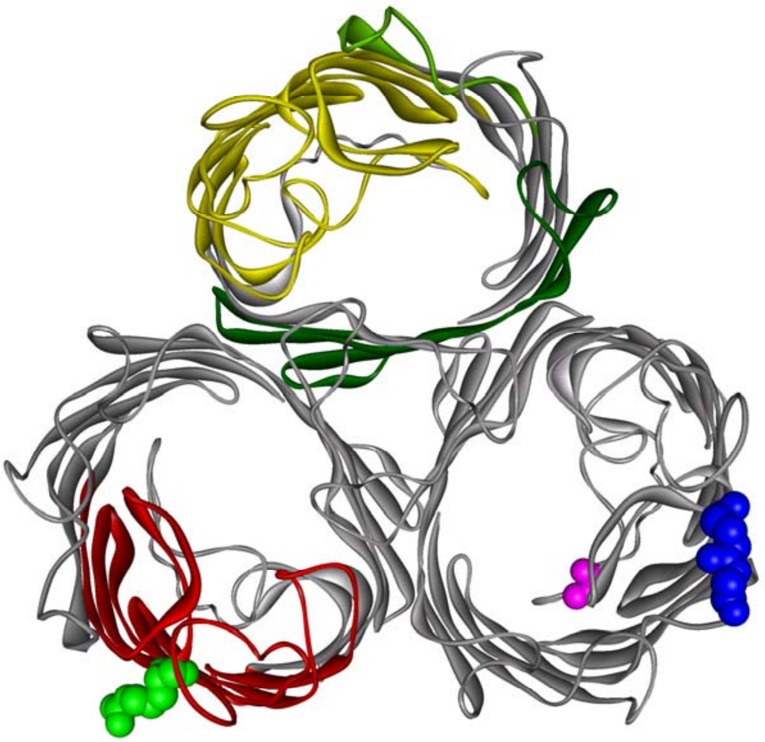

Figure S5. Colicin N Receptor and Translocator Regions of OmpF Identified by (Fourel *et al*., 1990)

Regions identified by use of OmpF-OmpC chimaeras shown as strands; Yellow and green highlight receptor binding; and Red translocation regions (space filling residue in green is Arg 235 in the suggested LPS binding site see figure 4). Residues identified by mutation to affect receptor binding, blue E284, G285 and magenta G119.

Supplemental References

Bainbridge, G., Armstrong, G.A., Dover, L.G., Whelan, K.F. and Lakey, J.H. (1998) Displacement of OmpF loop 3 is not required for the membrane translocation of colicins N and A *in vivo. FEBS Letters.*, **432**, 117-122.

Ferguson, A.D., Welte, W., Hofmann, E., Lindner, B., Holst, O., Coulton, J.W. and Diederichs, K. (2000) A conserved structural motif for lipopolysaccharide recognition by procaryotic and eucaryotic proteins. *Structure*, **8**, 585-592.

Fourel, D., Hikita, C., Bolla, J.M., Mizushima, S. and Pages, J.M. (1990) Characterization of ompF domains involved in Escherichia coli K-12 sensitivity to colicins A and N. *J Bacteriol*, **172**, 3675-3680.
